# Comparison of VerifyNow, thromboelastography, and PL-12 in patients with minor ischemic stroke or transient ischemic attack

**DOI:** 10.18632/aging.202650

**Published:** 2021-03-03

**Authors:** Lin Ma, Weiqi Chen, Yuesong Pan, Hongyi Yan, Hao Li, Xia Meng, Yongjun Wang, Yilong Wang

**Affiliations:** 1 Department of Neurology, Beijing Tiantan Hospital, Capital Medical University, Beijing, China; 2 China National Clinical Research Centre for Neurological Diseases (NCRC-ND), Beijing, China; 3 Advanced Innovation Centre for Human Brain Projection, Capital Medical University, Beijing, China

**Keywords:** high on-treatment platelet reactivity, platelet function, ishemic stroke, transient ischemic attack, antiplatelet therapy

## Abstract

High on-treatment platelet reactivity (HOPR) is associated with stroke recurrence. It is important to find a reliable method to assess HOPR. We aimed to compare the correlations between VerifyNow™ system, thromboelastography (TEG), and Aggrestar platelet function analyzer (PL-12) on platelet activity in patients with minor ischemic stroke or transient ischemic attack (TIA) after dual antiplatelet therapy for 7 days. About 276 patients were included. Spearman’s correlation coefficient and the kappa coefficient were adopted to evaluate associations among the three test methods. An obvious correlation between VerifyNow and TEG on HOPR-ADP (r=0.64, p<0.001) was found. The correlations of HOPR-ADP between PL-12 and the other two platelet function analyzers were moderate (PL-12 versus VerifyNow, r=0.47, p<0.001; PL-12 versus TEG, r=0.25, p<0.001). The correlations of HOPR-AA were limited among these three platelet function analyzers (VerifyNow versus TEG, r=0.09, p=0.14; VerifyNow versus PL-12, r=0.15, p=0.01; PL-12 versus TEG, r=0.10, p=0.09). Correlations among different platelet function analyzers were varied. VerifyNow and TEG were more correlative than PL-12 on HOPR-ADP. The consistence of HOPR-AA was limited among VerifyNow, TEG and PL-12. The proportion of stroke recurrence and composite events in patients with HOPR-ADP assessing by PL-12 was higher than VerifyNow and TEG.

## INTRODUCTION

Large clinical trials have shown that the combination of clopidogrel and aspirin reduced the risk of stroke recurrence [1, 2]. However, some patients still experience recurrent ischemic events with dual antiplatelet therapy. High on-treatment platelet reactivity (HOPR) refers to the limited degree of the inhibition of platelet aggregation compared with the inhibition expected using antiplatelet therapy [3]. HOPR is considered as one of the most important reasons of recurrent ischemic events with dual antiplatelet therapy [4, 5]. In this study, we used “High on-treatment platelet reactivity of AA” (HOPR-AA) and “High on-treatment platelet reactivity of ADP” (HOPR-ADP) to describe HOPR assessing by aspirin and P2Y12-specific cartridges respectively. It is valuable to assess HOPR in clinical practice [5–10].

Plenty of laboratory assays could be used to examine platelet function *in vitro*. Light transmission aggregometry (LTA) is a standard method for evaluating platelet function, which is based on the change of turbidity in light transmission. LTA is a flexible and time-consuming method. Its completion requires large blood volumes and experienced laboratory technicians [4, 7]. Quicker and more user-friendly analyzers are needed. VerifyNow™ system (VerifyNow) is a point-of-care platelet function analyzer based on light transmission, which is the same mechanism with LTA [8]. Previous studies have shown that VerifyNow got an obvious correlation with LTA [9, 11]. We consider VerifyNow as a standard analyzer in our study. Thromboelastography (TEG) is designed to measure clot formation, clot strength, and clot degradation, and it is one of the most common whole-blood platelet function tests used in clinical practice. However, TEG is subject to a unique set of pre-analytic and analytic variables that influence the reliability and reproducibility of the test [10]. Aggrestar platelet function analyzer (PL-12) is a new automated analyzer based on the platelet count drop method. It counts platelet twice before the addition of an agonist in whole blood samples [12]. However, the coagulation function, mean platelet volume, and number of platelets before the measurements might affect the interpretation of platelet reactivity assessed by PL-12.

Owing to the different detection principles, different methods have different advantages. Little research has been published about the agreement among VerifyNow, TEG, and PL-12 in patients with minor stroke or transient ischemic attack (TIA). Therefore, we aimed to compare TEG and PL-12 with VerifyNow to evaluate their agreement in assessing HOPR-AA/ADP in minor ischemic stroke or TIA patients, and to compare the correlation between HOPR assessed by platelet function analyzers and clinical events (i.e., stroke, TIA, myocardial infarction, or vascular death).

## RESULTS

### Baseline characteristics

Among 675 patients enrolled in the PRINCE trial, 276 patients were included in this analysis. The baseline characteristics of patients included and excluded in the subgroup analysis were shown in Supplementary Table 1. The median age of the participants included in the subgroup analysis was 61 years, and 28.9% of them were women. The index event was a minor stroke in 231 patients (83.7%) and a TIA in 45 patients (16.3%). The baseline laboratory characteristics were also compared between these two groups (Supplementary Table 1).

### Platelet function results

About 44 patients (15.94%) were detected to have HOPR-ADP via VerifyNow. TEG showed 39 patients (14.13%) with HOPR-ADP. About 14 patients (5.07) were detected to have HOPR-ADP by PL-12. Twenty-five patients (9.10%) showed HOPR-AA via VerifyNow. A number of 36 patients (13.04%) showed HOPR-AA via TEG, and 8 patients (2.90%) was found HOPR-AA in the PL-12.

### Comparison of different tests in assessing HOPR-ADP

**Figure 1:**
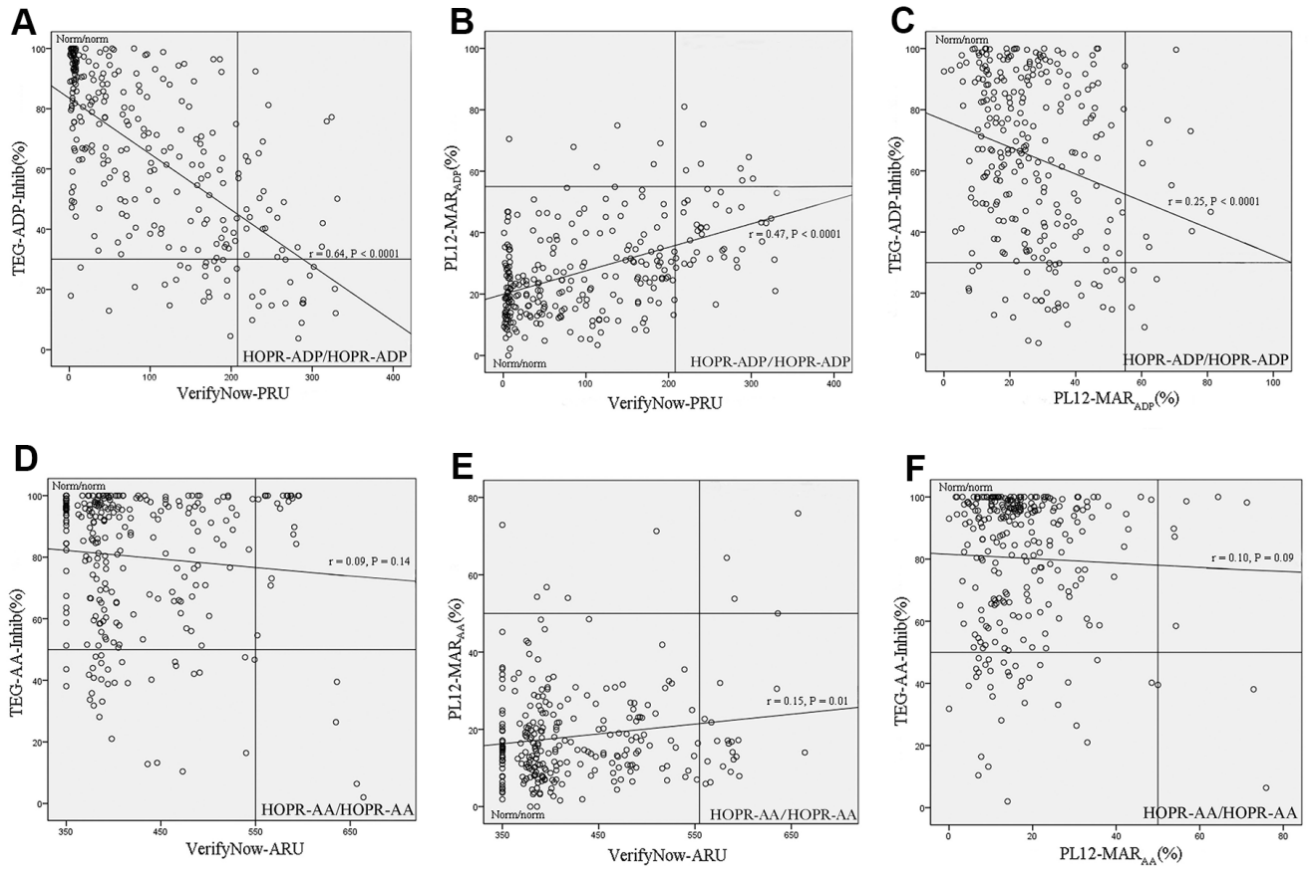
**Comparison of platelet function analyzers to assessing HOPR-ADP/AA.** (**A**) The comparison between VerifyNow and TEG in assessing HOPR-ADP. (**B**) The comparison between VerifyNow and PL-12 in assessing HOPR-ADP. (**C**) The comparison between TEG and PL-12 in assessing HOPR-ADP. (**D**) The comparison between VerifyNow and TEG in assessing HOPR-AA. (**E**) The comparison between VerifyNow and PL-12 in assessing HOPR-AA. (**F**) The comparison between TEG and PL-12 in assessing HOPR-AA.

The correlation between VerifyNow and TEG is highest in our study (r=0.64, p<0.001; Table 1). The kappa value is 0.31 (Table 2). The receiver operating characteristic (ROC) curve analysis suggests that 49.2% should be the cutoff value for PL-12, with 98% sensitivity and 16% specificity (Supplementary Table 2). A limited correlation was found between VerifyNow and TEG (r=0.09, p=0.14; Table 1), and the kappa value was 0.03 (Table 2). The ROC analysis suggested that the cutoff value of ADPI should be 58.8% for TEG, with a sensitivity of 68.1% and a specificity of 81.8% (Supplementary Table 2). The correlation between VerifyNow and PL-12 was also obvious (r=0.47, p<0.001; Table 1). The kappa value was 0.22 (Table 2). The cutoff value of MAR_ADP_ should be 28.6% with 95.5% sensitivity and 67.7% specificity (Supplementary Table 2). TEG and PL-12 showed a moderate correlation (r=0.25, p<0.001; Table 1). The kappa value was 0.08 (Table 3). The comparison of these three methods is shown in Figure 1.

**Table 1:** Correlation between VerifyNow, thromboelastography (TEG), and PL-12 for HOPR-ADP.

**Methods**	**PL-12**		**TEG**	
	**r**	**p Value**	**r**	**p Value**
VerifyNow	0.47	< 0.001	-0.64	< 0.001
TEG	-0.25	< 0.001		

Because HOPR-ADP in the TEG test was defined as ADP inhibition < 30%, which was contrary to the trends of other tests, the r value was negative.

**Table 2:** Comparison between VerifyNow, thromboelastography (TEG), and PL-12 in identifying HOPR.

**Method (agonist)**	**Definition of HOPR**	**Patients with HOPR n (%)**	**Kappa**
VerifyNow (ADP)	PRU > 208	44 (15.9)	- (reference method)
TEG (ADP)	ADPI < 30%	39 (14.1)	0.31
PL-12 (ADP)	MAR_ADP_ ≥ 55%	14 (5.1)	0.22
VerifyNow (AA)	ARU ≥ 550	25 (9.1)	- (reference method)
TEG (AA)	AAI < 50%	36 (13.0)	0.03
PL-12 (AA)	MAR_A_ > 50 %	8 (2.9)	0.14

HOPR, high on-treatment platelet reactivity; AA, arachidonic acid; ARU, aspirin reaction units; PRU, P2Y12 reaction units; AAI, arachidonic acid inhibition; ADPI, ADP inhibition; MAR_AA_, maximal platelet aggregation ratio of arachidonic acid-stimulated platelets; MAR_ADP_, maximal platelet aggregation ratio of ADP-stimulated platelets.

**Table 3:** Comparison between thromboelastography (TEG) and PL-12 in identifying HOPR.

**Method (agonist)**	**Definition of HOPR**	**Patients with HOPR n (%)**	**Kappa**
TEG (ADP)	ADPI < 30%	39 (14.1)	0.08
PL-12 (ADP)	MAR_ADP_ ≥ 55%	14 (5.1)	
TEG (AA)	AAI < 50%	36 (13.0)	0.05
PL-12 (AA)	MAR_A_ > 50 %	8 (2.9)	

HOPR, high on-treatment platelet reactivity; AA, arachidonic acid; AAI, arachidonic acid inhibition; ADPI, ADP inhibition; MAR_AA_, maximal platelet aggregation ratio of arachidonic acid-stimulated platelets; MAR_ADP_, maximal platelet aggregation ratio of ADP-stimulated platelets.

### Comparison of different tests in assessing HOPR-AA

HOPR-AA was limited among VerifyNow, TEG and PL-12. VerifyNow and PL-12 got a weak correlation (r=0.15, p=0.01; Table 4), and the kappa value was 0.14 (Table 2). The ROC analysis suggested that 49.2% should be the cutoff value for PL-12, with 98% sensitivity and 16% specificity (Supplementary Table 2). A limited correlation was found between VerifyNow and TEG (r=0.09, p=0.14; Table 4), and the kappa value was 0.03 (Table 2). The ROC analysis suggested that the cutoff value of TEG-AA inhibition should be 97.4% for PL-12, with 76% sensitivity and 56% specificity (Supplementary Table 2). The correlation between PL-12 and TEG was also limited (r=0.10, p=0.09; Table 4), and the kappa value was 0.05 (Table 3). The comparison of these three methods is shown in Figure 1.

**Table 4:** Correlation between VerifyNow, thromboelastography (TEG), and PL-12 for HOPR-AA.

**Methods**	**PL-12**		**TEG**	
	**r**	**p Value**	**r**	**p Value**
VerifyNow	0.15	0.01	-0.09	0.14
TEG	-0.10	0.09		

Because HOPR-AA in the TEG test was defined as arachidonic acid inhibition < 50%, which was contrary to the trends of other tests, the r value was negative.

### Relationships between HOPR detected by platelet function analyzers and clinical outcomes

Table 5 showed the relationship between HOPR and clinical outcomes. The proportions of stroke recurrence and composite events in patients with HOPR-ADP assessing by PL-12 were higher than VerifyNow and TEG. At 3 months, 2 patients (14.3%) who were found HOPR-ADP by VerifyNow got stroke recurrence and composite events, but no significant correlation was found (p=0.26). Compared with VerifyNow, TEG/PL-12 had lower proportion of stroke among patients with HOPA-ADP (VerifyNow: 13.6%, p=0.05; TEG: 7.7%, p=0.83). Two patients (25.00%) with HOPR-AA assessed by PL-12 were found stroke recurrence and composite events, and 2 (8.0%) were found in VerifyNow. TEG found that three patients (8.3%) with HOPR-AA got recurrent stroke.

**Table 5:** The association between HOPR and clinical outcomes in patients with HOPR monitored by VerifyNow, TEG and PL-12.

		**Stroke recurrence**		**Composite events**	
		**N (%)**	**P value**	**N (%)**	**P value**
HOPR-ADP	VerifyNow	6 (13.6)	0.05	6 (13.6)	0.13
	TEG	3 (7.7)	0.83	3 (7.7)	0.94
	PL-12	2 (14.3)	0.26	2 (14.3)	0.37
HOPR-AA	VerifyNow	2 (8.0)	0.82	2 (8.0)	0.99
	TEG	3 (8.3)	0.71	3 (8.3)	0.93
	PL-12	2 (25.0)	0.04	2 (25.0)	0.07

HOPR-AA, high on-treatment platelet reactivity of AA; HOPR-ADP, high on-treatment platelet reactivity of ADP.

## DISCUSSION

We compared three platelet function assays (VerifyNow, TEG and PL-12) for assessing HOPR-AA/ADP in patients with minor ischemic stroke or TIA. Both TEG and PL-12 got an obvious correlation with VerifyNow in monitoring HOPR-ADP. However, the correlation of HOPR-ADP between PL-12 and TEG is moderate. VerifyNow got little correlation with TEG and PL-12. The correlation between PL-12 and TEG is also limited.

Previous studies evaluating the platelet function found a very low prevalence of HOPR-AA, and such results may likely be due to noncompliance [1, 13]. Our results were in agreement with these observations. The small numbers of HOPR-AA made this comparison more uncertain [14–18]. Large cohort studies were needed to confirm the correlation of HOPR-AA between these three platelet function tests. In our study, the prevalence of HOPR-ADP was higher than previous study. Madsen et al. compared VerifyNow and TEG with LTA for assessing the long-term effects of concomitant aspirin and clopidogrel therapy on platelet inhibition in patients treated with elective PCI [5]. A number of 33 patients completed tests at baseline. Four patients were found HOPR-AA in the LTA test, and both TEG and VerifyNow found only one patient who got HOPR-AA. The results of our study might be attributed to the fact that HOPR-ADP were more popular in Chinese population [19]. And the sample size of our analysis was larger than previous studies. Further, the sample size of our analysis was larger than that of previous negative studies.

The consistence between platelet function analyzers in previous studies was varied. Some studies reported that VerifyNow and TEG were moderate or non-correlative in assessing HOPR-ADP [1, 13, 15], whereas other studies found obvious correlations. Guan et al. demonstrated an obvious correlation between PL-11 with VerifyNow and TEG in health individuals [12]. Correlations of HOPR-ADP between methods were obvious (PL-11 versus VerifyNow, r=0.83, p<0.01; PL-11 versus TEG, r=0.70, p<0.001). In our study, we assessed HOPR-AA and HOPR-ADP after dual antiplatelet therapy for 7 days. Dual antiplatelet therapy was more effective at 7 days than baseline, and the result of platelet function was more reliable.

Previous studies have investigated HOPR was associated with stroke recurrence. However, few studies compared the correlation between HOPR assessed by different platelet function analyzers and clinical outcomes. In our studies, we found the proportion of stroke recurrence was highest in patients with HOPR monitored by PL-12. It might suggested that PL-12 was more reliable to predict stroke recurrence in clinical practice.

Difference analyzers have difference advantage and disadvantage. Verify Now is a commonly used point-of-care platelet function test. It uses optical turbidimetric technology to evaluate the platelet function [20]. However, erythrocytes may influence the signal during detection, which may cause bias. TEG analyzes the movement of the wire in the blood sample and yields the maximal clot strength, which is graphically displayed on the TEG trace [21]. PL-12 is a new automated point-of-care platelet function analyzer that is different from the two other methods. It counts platelet twice before and more than trice after the addition of an agonist in the same citrated whole blood samples. PL-12 correlated well with VerifyNow, and also correlated with TEG on HOPR-ADP in this analysis. In our study, the proportion of stroke recurrence in patients with HOPR assessed by PL-12 was higher than VerifyNow and TEG. It reminded that PL-12 offered a standardized operation for platelet count drop method.

There were several advantages in our study. First, we analyzed HOPR after dual antiplatelet drug for 7 days. Dual antiplatelet therapy effected at 7 days, and the platelet reactivity were more reliable. Second, we analyzed HOPR among patients with minor stroke or TIA. Little research has reported HOPR in patients with stroke or TIA. Coagulation functions, haemodynamic, and pathogenesis were different between stroke patients and other disease patients, which may influence the results of HOPR. Third, we investigated the relationship between HOPR and stroke recurrence among three platelet function analyzers.

Our study has several limitations. First, the number of HOPR-AA was small, which may influence the comparison of HOPR-AA. Much further large clinical and experimental studies are required. Second, we used VerifyNow as standard method in point-of-care platelet function analyzers. Future studies involving LTA and other platelet function tests are needed. Third, some factors might affect the interpretation of platelet reactivity monitoring by platelet function tests in our study, such as the coagulation function and the time between collecting blood samples and testing. Fourth, the HOPR of Chinese stroke patients are different from that in European patients. The results of our study should be evaluated in different populations in the future.

In conclusion, compared to VerifyNow, TEG showed a better correlation than PL-12 in assessing HOPR-ADP. PL-12 and TEG got a moderate correlation in assessing HOPR-ADP. The prevalence of HOPR-AA was low, and the correlations between VerifyNow, TEG and PL-12 were limited. Compared with VerifyNow/TEG, the proportion of stroke recurrence in HOPR-ADP assessing by PL-12 was higher.

## MATERIALS AND METHODS

### Overview of the PRINCE trial and the platelet function test substudy

**Figure 2:**
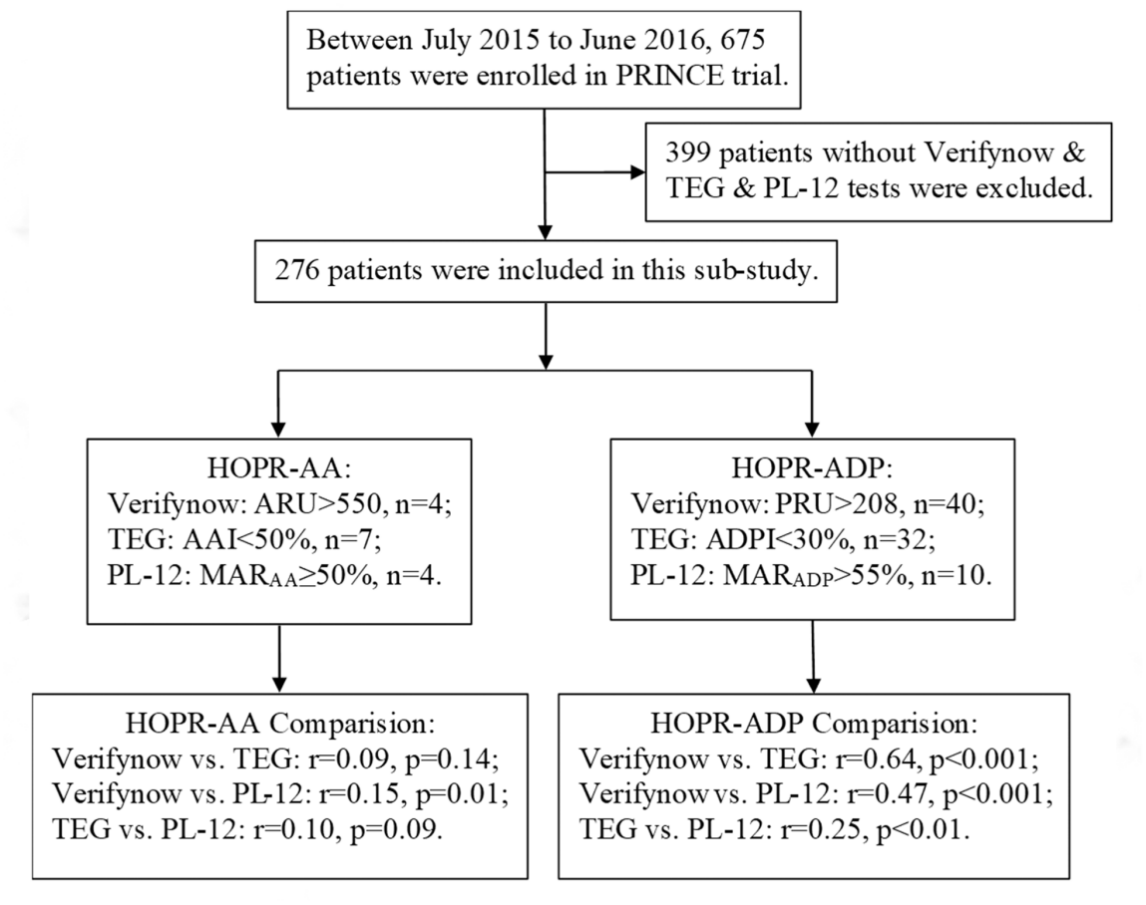
**Flow diagram for the enrollment process of this study from the PRINCE trial.**

The PRINCE (Effect of Ticagrelor with Clopidogrel on High On-treatment Platelet Reactivity in Acute Stroke or Transient Ischemic Attack) trial was designed as a prospective, multicenter, randomized, open-label, active-controlled, and blind-endpoint, phase IIb trial [1, 22]. The details of the design, rationale, and major results have been previously described. The data of the present subgroup analysis were derived from the prespecified platelet function test substudy of the PRINCE trial. The substudy involved three visits: 2 hours after taking the first agents, 24 hours after taking the first agents, and day 7 + 2 days. The blood samples of this subgroup were collected at 2–4 hours after taking the investigational drugs. Patients without all three platelet function tests at 7 days were excluded from the present analysis. Of the 26 centers included in the PRINCE trial, a total of 276 participants in 11 centers voluntarily participated in this analysis, in which the platelet reactivity was evaluated by VerifyNow, TEG, and PL-12 (Figure 2).

### Standard protocol approvals, registrations, and patient consents

The PRINCE trial was registered in ClinicalTrials.gov (NCT02506140) and approved by the ethics committee of Beijing Tiantan Hospital and all centers. Written informed consent was obtained from all participants or their legal representatives before being entered into the study.

### Blood sampling

Six tubes of peripheral venous blood samples were collected at 2–4 hours after taking the investigational drugs. The first 2 mL of blood was discarded. A 3.2% sodium citrate tube (Greiner Bio-One Vacuette North America Inc., Monroe, NC) was used for VerifyNow analysis. A heparin tube (Becton-Dickinson, Franklin Lakes, NJ) was used for the TEG test, and two 3.2% sodium citrate tubes (Becton-Dickinson, Franklin Lakes, NJ) were used for the TEG and PL-12 tests, respectively. The tubes were gently inverted 10 times to ensure complete mixing of the blood sample with the anticoagulant. Blood samples were kept at 27° C before testing. The whole procedure needed to be performed within 2 hours after sampling.

### Platelet function tests

### VerifyNow analysis

The VerifyNow system (Accumetrics, San Diego, CA, USA) is a point-of-care test based on the optical change in whole blood samples to identifying platelet function [4, 23]. The whole blood sample was decanted into the reaction cartridge after being inserted into the apparatus. We used the aspirin- and P2Y12-specific cartridges to identify platelet dysfunction caused by aspirin and clopidogrel, respectively. Arachidonic acid (AA) was the platelet activation agent used in the aspirin test. If aspirin was not effective, cyclooxygenase-1 was activated to transform AA to thromboxane A2, leading to platelet aggregation. The degree of aggregation is reported in aspirin reaction units (ARU). A value ≥ 550 ARU indicates HOPR-AA [24, 25]. In the P2Y12-specific cartridge, the P2Y12 receptor was specially suppressed by ADP, and the changes in light transmission was measured as P2Y12 reaction units (PRU). A value > 208 PRU was defined as HOPR-ADP [23, 26, 27].

### TEG platelet mapping™ assay

TEG (Heamoscope Corporation, Niles, IL) is a noninvasive assay that tests platelet function [28]. Different agonists and anticoagulant venous blood samples were placed into a special cup. The concentration of the activator added to the whole blood was 1 mmol/L AA or 2 mmol/L ADP. A special cup containing venous blood was rotated at 4° 45′, with each rotation lasting 10 s. The fibrin-platelet complex cohered to the cup, and the rotary torque of the cup was transferred to the pin immersed in the blood sample. The rotation of the pin was converted to electrical signals by electromechanical sensors, which were monitored by a computer. Maximum amplitude (MA) directly reflects the maximal clot strength that facilitates the formation of the cross-linked fibrin clot. The MA of the cyclooxygenase-1 pathway and P2Y12 was measured as MA_AA_ and MA_ADP_, respectively. We defined a<50% and<30% inhibition of AA- and ADP-induced clot formation (i.e., TEG-AA inhibition, TEG-ADP inhibition [ADPI]) as HOPR-AA and HOPR-ADP, respectively [6, 29]. AA/ADPI was calculated using the following formula:

$$\text{AA}\;\text{or}\;\text{ADP}\;\text{Inhibition}=100-100\times \left[\frac{\left({\text{MA}}_{\text{AA or ADP}}-{\text{MA}}_{\text{fibrin}}\right)}{\left({\text{MA}}_{\text{thromb}}-{\text{MA}}_{\text{fibrin}}\right)}\right]$$

### PL-12 analysis

The PL-12 platelet function analyzer (SINOWA Medical Science and Technology Co., Nanjing, China) is a new point-of-care platelet function analysis tool based on the SPCM [12]. SPCM was used to determine platelet parameters such as number and volume, and to calculate the platelet aggregation rate by using the change of platelet number before and after the agent was induced, thus allowing to dynamically evaluate the platelet function. The blood sample was gently mixed in a constant temperature for 10 min. Thereafter, 500 mL citrated blood sample was transferred to the detecting position. The whole analysis procedure was performed automatically. When the aggregated platelets were too large to be counted, they were dropped from the single platelet counting. PL-12 counted several times until the lowest level was detected. A value of MAR_AA_ > 50% was defined as HOPR-AA, and a value of MAR_ADP_ ≥ 55% was defined as HOPR-ADP. The maximal platelet aggregation ratio (MAR) was calculated using the following formula:

$$\begin{array}{l} \text{Percentage}\;\text{of}\;\text{MAR}\;\left(\%MAR\right)=\\ \{100-[\frac{\left(1^{\text{st}}\;\text{platelet count}+2^{\text{nd}}\text{platelet count}\right)}{2}\\ -\text{lowest platelet count}]\}\times 100\% \end{array}$$

### Outcomes assessment

The study’s primary outcome was the proportion of patients with HOPR at 7 days. Secondary outcomes were clinical outcomes at 90 ± 7 days. The clinical outcomes included ischemic stroke and composite clinical vascular events (ischemic/hemorrhagic stroke, TIA, myocardial infarction, or vascular death) at 90 ± 7 days.

### Statistical analysis

Continuous variables are presented as means with standard deviations or medians with interquartile ranges, and categorical variables are presented as percentages. The baseline characteristics were compared between the included group and the excluded group, using Student’s t-test or the Wilcoxon test for continuous variables, and the χ^2^ test for categorical variables. Kappa analysis was used to assess the classification consistency of HOPR among the three methods. Pearson’s correlation coefficient was adopted to evaluate the relationships among methods when the data were in a normal distribution. The χ^2^ test was used to compared HOPR and clinical outcomes. Two-sided p values<0.05 were considered statistically significant. All analyses were performed using SAS 9.4 (SAS Institute, Cary, NC, USA). Anonymized data are available to researchers on request for reproducing the results or replicating the procedures by contacting the corresponding author.

## Supplementary Material

Supplementary Tables
