# Supplementary material for: Comparison of VerifyNow, thromboelastography, and PL-12 in patients with minor ischemic stroke or transient ischemic attack
Source: Aging (Albany NY). 2021 Mar 3;13(6):8396–407. doi: 10.18632/aging.202650 (PMC8034945; doi:10.18632/aging.202650)
Supplement: Supplementary Tables [file aging-13-202650-s001.pdf]

SUPPLEMENTARY TABLES

Supplementary Table 1. Baseline characteristics of patients with HOPR monitored by verifynow, TEG and PL-12 at 7 days and patients without all these tests.

Characteristic	Patients with HOPR monitored by verifynow, TEG and PL-12 (n = 276)	Patients without HOPR monitored by verifynow, TEG and PL-12 (n = 399)	p Value
Age (years)	61.2 ± 8.8	61.2 ± 8.7	0.27
Median	61.0	62.0	
Interquartile range	55.5–67.0	54.0–67.0	
Female sex, no. (%)	195 (28.89)	299 (44.30)	0.22
Medical history, no. (%)			
Hypertension	166 (24.59)	245 (36.30)	0.74
Dyslipidemia	21 (3.11)	20 (2.96)	0.17
Diabetes mellitus	60 (8.89)	104 (15.41)	0.20
Ischemic stroke	50 (7.41)	71 (10.52)	0.91
TIA	9 (1.33)	9 (1.33)	0.43
Coronary artery disease	7 (1.04)	44 (6.52)	< 0.001
Smoking status, no. (%)			0.06
Nonsmoker	131 (19.41)	174 (25.78)	
Current smoker	118 (17.48)	201 (29.78)	
Ex-smoker	27 (4.00)	24 (3.56)	
Drug use before randomization — no. (%)			
Statin	37(5.48)	29(4.30)	0.01
Aspirin	68(10.07)	78(11.56)	0.11
Clopidogrel	9(1.33)	6(0.89)	0.12
Qualifying event, no. (%)			0.93
Minor stroke	231 (34.22)	333 (49.33)	
TIA	45 (6.67)	66 (9.78)	
Platelet count (10^9/L)	217.5 ± 60.1	220.0 ± 58.8	0.60
Median	210.0	216	
Interquartile range	177.0–252.0	178.0–255.0	
APTT (s)	32.7 ± 5.4	32.5 ± 5.4	0.69
Median	33.1	32.6	
Interquartile range	29.2–36.4	29.2–35.7	
VerifyNow			
PRU	259.8 ± 55.6	246.1 ± 58.7	0.002
Median	260.0	243.0	
Interquartile range	224.0–296.0	210.0–277.0	
ARU	584.4 ± 93.8	575.6 ± 96.9	0.24
Median	637.0	627.0	
Interquartile range	506.0–657.0	494.5–653.5	

BMI, body mass index; TIA, transient ischemic attack; APTT, activated partial thromboplastin time; PRU, P2Y12 reaction units; ARU, aspirin reaction units.

Supplementary Table 2. Test performance characteristics with receiver operating characteristic curve analysis.

	AUC	95% CI	Sensitivity	Specificity	Cutoff value
TEG					
AAI	0.62	0.48–0.76	56.0%	76.0%	> 97.4%
ADPI	0.81	0.75–0.87	68.1%	81.8%	< 58.8%
PL-12					
MAR_{AA}	0.54	0.42–0.66	16%	98.0%	> 49.2%
MAR_{ADP}	0.84	0.79–0.90	95.5%	67.7%	≥ 28.6%

AUC, area under the curve; CI, confidence interval; TEG, thromboelastography; AAI, arachidonic acid inhibition, the percentage inhibition of the contribution from arachidonic acid-stimulated platelets to maximal clot strength (TEG assay); ADPI, ADP inhibition, the percentage inhibition of the contribution from ADP-stimulated platelets to maximal clot strength (TEG assay); MAR_{AA}: maximal platelet aggregation ratio of arachidonic acid-stimulated platelets (PL-12); MAR_{ADP}: maximal platelet aggregation ratio of ADP-stimulated platelets (PL-12).
